# The feasibility of *in vivo* imaging of infiltrating blood cells for predicting the functional prognosis after spinal cord injury

**DOI:** 10.1038/srep25673

**Published:** 2016-05-09

**Authors:** Kazuya Yokota, Takeyuki Saito, Kazu Kobayakawa, Kensuke Kubota, Masamitsu Hara, Masaharu Murata, Yasuyuki Ohkawa, Yukihide Iwamoto, Seiji Okada

**Affiliations:** 1 Department of Orthopaedic Surgery, Graduate School of Medical Sciences, Kyushu University, 3-1-1 Maidashi, Higashi-ku, Fukuoka, 812-8582, Japan; 2 Department of Advanced Medical Initiatives, Graduate School of Medical Sciences, Kyushu University, 3-1-1 Maidashi, Higashi-ku, Fukuoka 812-8582, Japan; 3 Department of Transcriptomics, JST-CREST, Medical Institute of Bioregulation, Kyushu University, 3-1-1 Maidashi, Higashi-ku, Fukuoka 812-8582, Japan

## Abstract

After a spinal cord injury (SCI), a reliable prediction of the potential functional outcome is essential for determining the optimal treatment strategy. Despite recent advances in the field of neurological assessment, there is still no satisfactory methodology for predicting the functional outcome after SCI. We herein describe a novel method to predict the functional outcome at 12 hours after SCI using *in vivo* bioluminescence imaging. We produced three groups of SCI mice with different functional prognoses: 50 kdyn (mild), 70 kdyn (moderate) and 90 kdyn (severe). Only the locomotor function within 24 hours after SCI was unable to predict subsequent functional recovery. However, both the number of infiltrating neutrophils and the bioluminescence signal intensity from infiltrating blood cells were found to correlate with the severity of the injury at 12 hours after SCI. Furthermore, a strong linear relationship was observed among the number of infiltrating neutrophils, the bioluminescence signal intensity, and the severity of the injury. Our findings thus indicate that *in vivo* bioluminescence imaging is able to accurately predict the long-term functional outcome in the hyperacute phase of SCI, thereby providing evidence that this imaging modality could positively contribute to the future development of tailored therapeutic approaches for SCI.

Traumatic spinal cord injury (SCI) causes permanent motor/sensory dysfunction, resulting in a marked reduction in the quality of life^1^. While spontaneous recovery of the motor function in patients with motor-complete SCI is limited, recovery in patients with incomplete SCI is more substantial and highly variable^2^,^3^. A reliable prediction of a patient’s potential functional outcome is therefore essential for counseling the patients and designing specific rehabilitation programs in accordance with the expected outcome.

There have been several attempts to establish the methods for predicting the functional outcome after SCI. First, serum biochemical markers such as phosphorylated neurofilament heavy chain subunit (pNF-H), S100 calcium-binding protein B (S100β), glial fibrillary acidic protein (GFAP) and microRNAs were shown to be useful for predicting the extent of neural damage^4^,^5^,^6^. Second, imaging analyses using magnetic resonance imaging (MRI) or diffusion tensor imaging (DTI) made it possible to visualize the lesions after SCI^7^,^8^. Third, neurophysiological assessments such as motor evoked potentials (MEP), somatosensory evoked potentials (SSEP) and electromyography (EMG) could be used to evaluate axonal tract conduction or muscle activity^9^,^10^. Despite these advances in the field of neurological assessment, no methodology has been able to satisfactorily predict the functional outcome after SCI.

*In vivo* bioluminescence imaging with firefly luciferase is a powerful tool for non-invasively visualizing cellular localization in living mice. This imaging method is based on the finding that firefly luciferase chemically produces light from a small-molecule substrate, D-luciferin^11^. The inherent simplicity and high sensitivity of this imaging method has led to *in vivo* monitoring of cellular dynamics in such processes as the proliferation or metastasis of cancer cells and the engraftment of transplanted stem cells^12^,^13^. We hypothesized that the number of blood-derived infiltrating cells is dependent on the severity of SCI, and this bioluminescence imaging method would enable us to quantify these infiltrating cells in the injured spinal cord. The overall aim of this study was to determine whether we could predict the functional outcome in the acute phase of SCI according to the severity of the injury as assessed by *in vivo* bioluminescence imaging.

In the present study, we produced three experimental mouse SCI models, each of which had different functional outcomes. To detect the bioluminescence signals derived from infiltrating cells in the injured spinal cord, we transplanted luciferase-positive bone marrow cells into lethally irradiated wild-type mice. We observed that the functional outcome after SCI correlated with the number of infiltrating neutrophils, and the number of infiltrating neutrophils had a strong proportional relationship to the bioluminescence signal intensity at 12 hours after SCI. These findings show that *in vivo* bioluminescence imaging is a potentially useful method for predicting the functional outcome after SCI.

## Results

### Differences in the functional prognosis after mild, moderate and severe SCI

**Figure 1:**
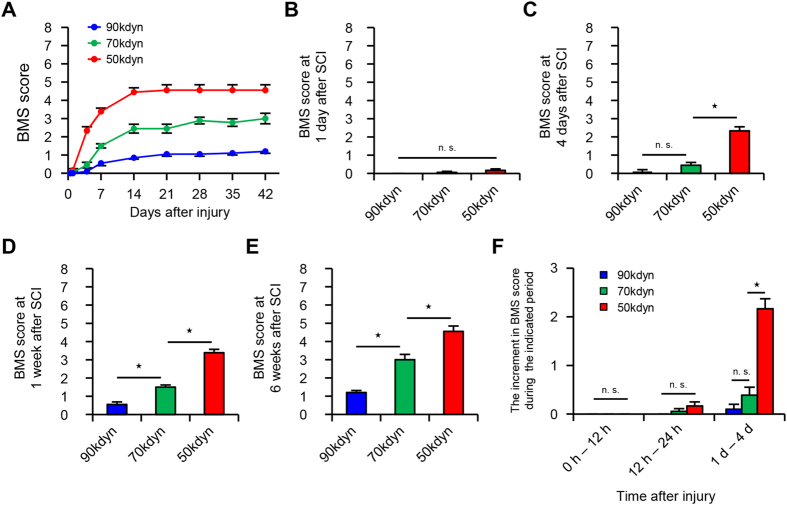
The different functional prognoses after mild, moderate and severe SCI. (**A**) The time course of functional recovery according to the BMS score in the mild (50 kdyn), moderate (70 kdyn) and severe (90 kdyn) SCI groups (n = 10–12 mice per group). (**B–E**) The BMS score from 1day to 6 weeks after SCI in the mild (50 kdyn), moderate (70 kdyn) and severe (90 kdyn) SCI groups. *P < 0.05, two-way repeated-measures analysis of variance (ANOVA) with the Tukey-Kramer post-hoc test, n.s. = not significant (P > 0.05), ANOVA (n = 10–12 mice per group). (**F**) The time-dependent functional recovery according to the BMS score during 0 to 12 hours, during 12 to 24 hours, and during 1 to 4 days after SCI in the mild (50 kdyn), moderate (70 kdyn) and severe (90 kdyn) SCI groups *P < 0.05, two-way repeated-measures analysis of variance (ANOVA) with the Tukey-Kramer post-hoc test, n.s. = not significant (P > 0.05), ANOVA (n = 10–12 mice per group). The data are presented as the means ± SEM.

In order to develop SCI mouse models with different functional prognoses, we first produced three groups of mice with injuries of different levels of severity: 50 kdyn (mild), 70 kdyn (moderate) and 90 kdyn (severe). Although we hardly observed any hindlimb movement in all three groups at 12 hours after injury, spontaneous recovery of the three groups varied during the first month after SCI as the time after the injury passed (Fig. 1A). Even at 24 hours after SCI, all three groups of SCI mice hardly presented spontaneous recovery (Fig. 1B). At 4 days after SCI, a significant difference in the locomotor function score (BMS score) was observed in the mild SCI group compared to those in the moderate and severe SCI groups (Fig. 1C). At 1 and 6 weeks after SCI, the locomotor function score among the three groups was significantly different from one another (Fig. 1D,E). The degree of spontaneous recovery correlated with the severity of the injury in the chronic phase of SCI (Fig. 1E), thus showing that our method reliably created groups of mice with different functional outcomes. To test whether we could predict the long-term functional outcomes of these SCI mice by assessing the functional changes within 4 days after SCI, we measured the difference in the locomotor function scores between 0–12 hours, between 12–24 hours and between 1–4 days after SCI. Whereas the functional recovery in all three groups was comparable between 0–12 hours and 12–24 hours, the mild SCI group showed a significantly better functional recovery between 1–4 days after SCI in comparison to the moderate and severe SCI groups (Fig. 1F). These results indicate that only an assessment of the locomotor function (BMS score) at least within 24 hours after SCI is not suitable for predicting the subsequent recovery potential after SCI. An alternative method is thus necessary for predicting the long-term functional outcome before 24 hours after SCI.

### Neutrophils infiltrate the injured spinal cord immediately after SCI

**Figure 2:**
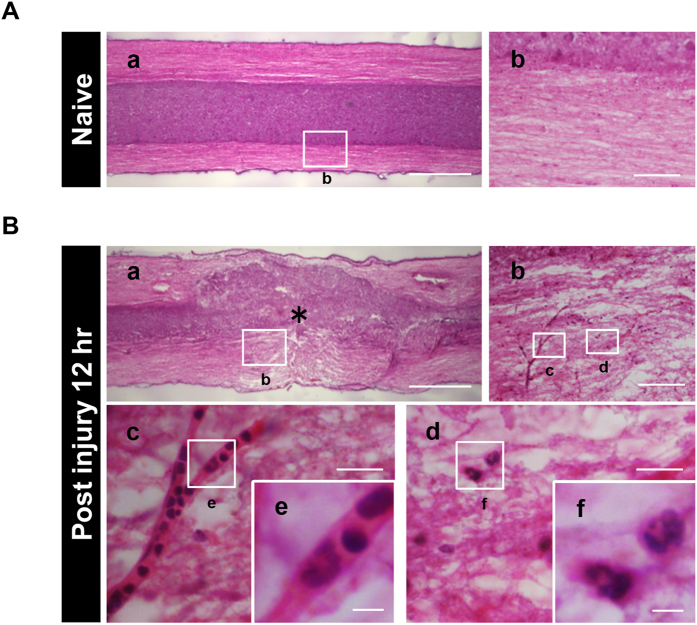
The structural integrity of the spinal cord is disrupted after SCI. (**A**) Hematoxylin and eosin stained sagittal sections of the naive spinal cord showed the integrity of the neural structure, as well as a distinct demarcation between the white matter and gray matter. The image in (**A**–b) is a magnification of the boxed area in (**A**–a). (**B**) The neuronal structure was disrupted at 12 hours after SCI as shown by hematoxylin and eosin staining. The images in (**B**–b), (**B**–c), (**B**–d), (**B**–e) and (**B**–f) are magnifications of the boxed areas in (**B**–a), (**B**–b), (**B**–b), (**B**–c) and (**B**–d), respectively. The asterisk indicates the epicenter of the lesion. The images in (**B**–c) and (**B**–d) showed the blood vessel lumen and the characteristic lobulated nuclei of inflammatory cells. Scale bars (**A**–a,**B**–a): 500 μm; (**A**–b,**B**–b): 200 μm; (**B**–c,**B**–d): 50 μm; (**B**–e,**B**–f): 10 μm.

To clarify the pathohistological changes before detecting functional differences among the three SCI groups, we performed a histological analysis using sagittal sections of the injured spinal cord at 12 hours after SCI (Fig. 2A,B). Hematoxylin and eosin staining showed the integrity of the neural structures in the naive spinal cord (Fig. 2A); however, at 12 hours after SCI, the integrity was remarkably disrupted and there was no distinct demarcation between the white matter and gray matter (Fig. 2B). In addition, we found a large number of polymorphonuclear cells inside or outside the blood vessel lumen in the injured spinal cord, whereas these polymorphonuclear cells were only rarely observed in the naive spinal cord. These findings suggest that non-neural cells infiltrate the injured spinal cord through the blood vessels at 12 hours after SCI.

**Figure 3:**
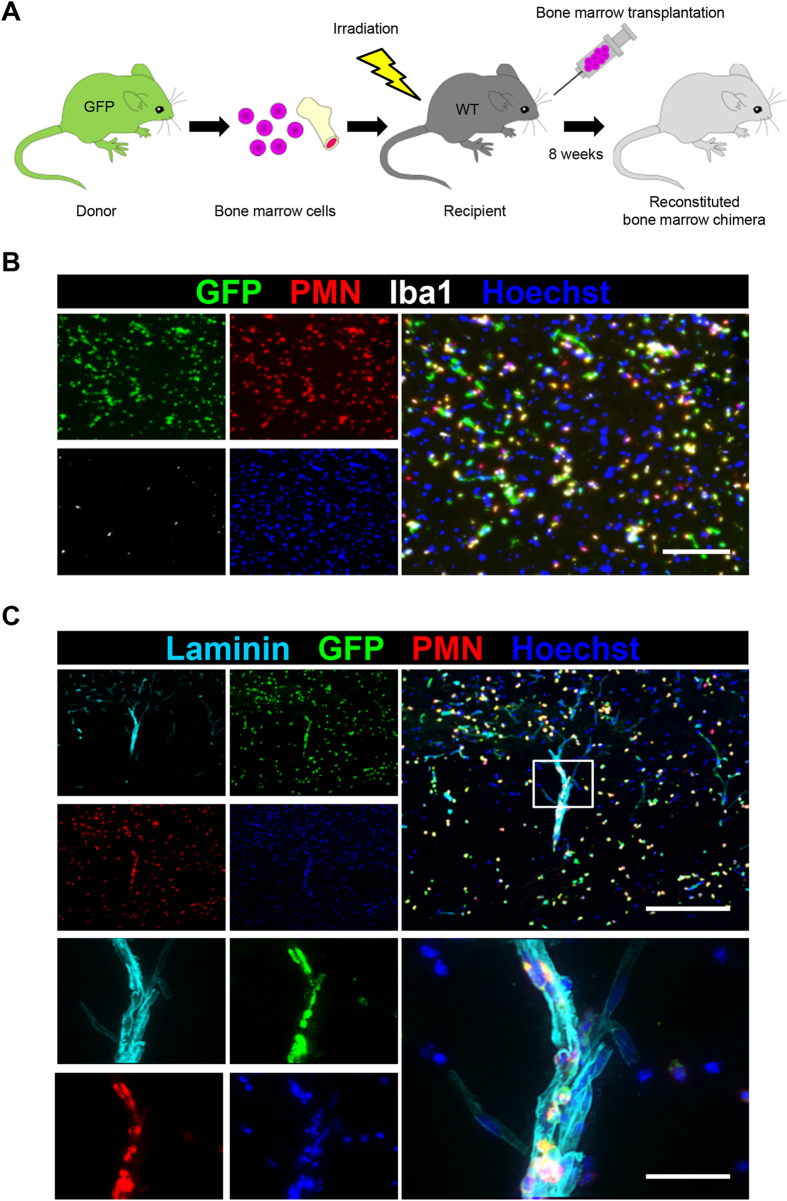
Peripheral blood-derived neutrophils infiltrate the injured spinal cord after SCI. (**A**) A schematic illustration of the generation of bone marrow chimeric mice. Whole bone marrow was harvested from a green fluorescence protein (GFP) transgenic mouse donor. Bone marrow cell transplantation was performed, and the reconstituted bone marrow chimeric mice were analyzed at eight weeks after transplantation. (**B**) The immunohistochemical analysis of the injured spinal cord at 12 hours after SCI with GFP (green), PMN (red), Iba1 (white) and Hoechst (blue) staining. Almost all of the infiltrating peripheral blood-derived cells were PMN-positive neutrophils. (**C**) An immunohistochemical analysis of the injured spinal cord at 12 hours after SCI with laminin (light blue), GFP (green), PMN (red) and Hoechst (blue) staining. The lower images are magnifications of the boxed areas in the upper images. Neutrophils were observed inside the laminin-positive blood vessel walls. Scale bars (**B**): 100 μm; (**C**): 200 μm; inset: 50 μm.

Previous studies have shown that the blood cell infiltration peaks at 12 hours after SCI and gradually decreases thereafter^14^,^15^,^16^. We thus speculated that the infiltrating cells were derived from blood cells such as neutrophils and monocytes/macrophages. To clarify the cell type and the distribution of blood-derived cells in the injured spinal cord, we generated bone marrow chimeric mice by transplanting bone marrow cells from mice that ubiquitously expressed green fluorescence protein (GFP) into lethally irradiated wild-type mice (Fig. 3A). Using these chimeric mice, we performed histological analyses of the injured spinal cord at 12 hours after SCI. Fluorescence microscopy showed the infiltration of GFP-positive blood-derived cells in the injury site at 12 hours after SCI. In addition, immunofluorescence staining of blood-derived cells demonstrated that GFP-positive/PMN-positive neutrophils markedly infiltrated the injured spinal cord during 12 hours after SCI, whereas we rarely observed GFP-positive/Iba1-positive monocytes/macrophages during 12 hours after SCI (Fig. 3B). The result that neutrophils markedly infiltrate the injured spinal cord at 12 hours after SCI is consistent with previous reports^14^,^16^. As expected, these GFP-positive/PMN-positive infiltrating neutrophils were observed along the surface of laminin-positive blood vessels (Fig. 3C). Newly formed blood vessels were rarely observed within 12 hours after SCI (Fig. S1). These results support the notion that neutrophils infiltrate the injury site through the remaining blood vessels.

### Differences in the number of infiltrating neutrophils after mild, moderate and severe SCI

**Figure 4:**
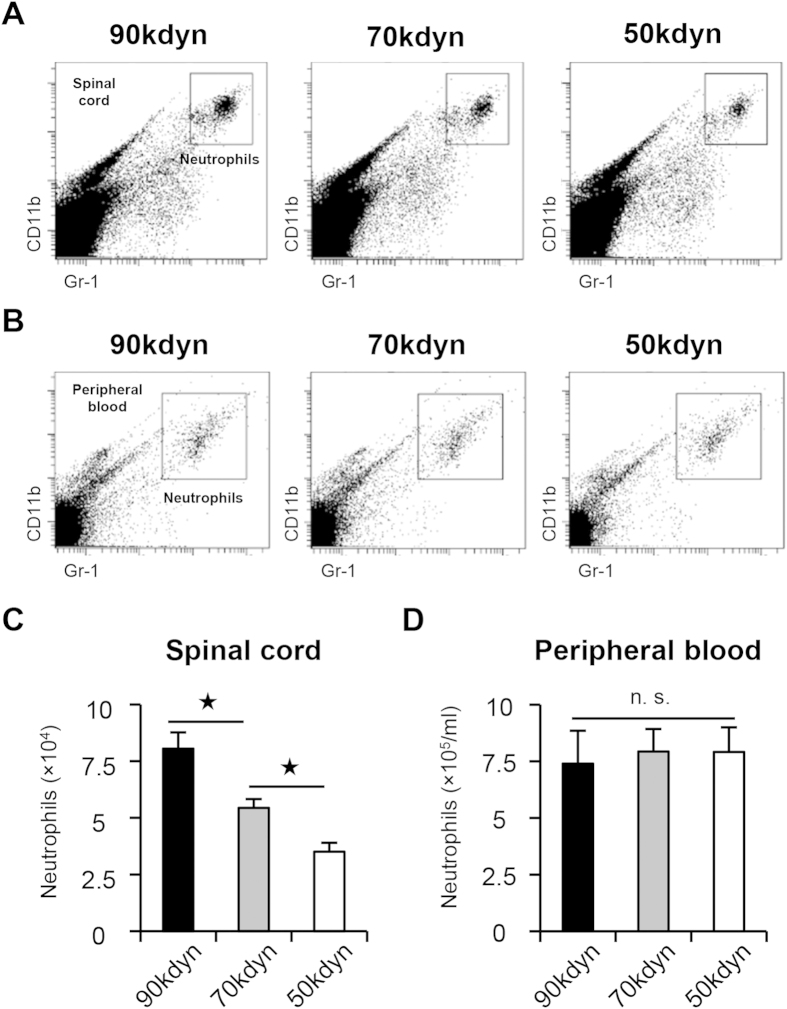
The quantification of the number of neutrophils in the injured spinal cord and in the peripheral blood after SCI. (**A,B**) Flow cytometry showed CD11b^high^/Gr-1^high^ neutrophil fractions in the injured spinal cord (**A**) and the peripheral blood (**B**) at 12 hours after SCI in the mild (50 kdyn), moderate (70 kdyn) and severe (90 kdyn) SCI groups. (**C**) The comparison of the number of infiltrating neutrophils in the injured spinal cord at 12 hours after SCI in the mild (50 kdyn), moderate (70 kdyn) and severe (90 kdyn) SCI groups, as determined by flow cytometry. *P < 0.05, ANOVA with the Tukey-Kramer post-hoc test (n = 7 mice per group). (**D**) The number of neutrophils was comparable in the peripheral blood of the mild (50 kdyn), moderate (70 kdyn) and severe (90 kdyn) SCI groups, even after SCI. n.s. = not significant (P > 0.05), ANOVA (n = 7 mice per group).

**Figure 5:**
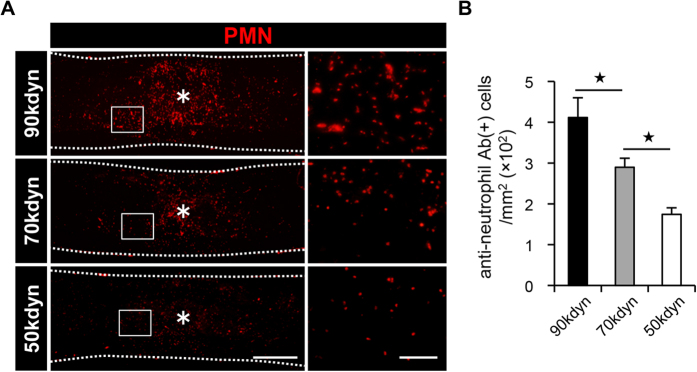
The number of infiltrating neutrophils in the injured spinal cord correlates with the severity of SCI. (**A**) An immunohistochemical analysis of the injured spinal cord at 12 hours after SCI with PMN (red) staining in the mild (50 kdyn), moderate (70 kdyn) and severe (90 kdyn) SCI groups. The asterisk indicates the epicenter of the lesion. The right images are magnifications of the boxed areas in the left images. (**B**) The comparison of the number of infiltrating neutrophils in the injured spinal cord at 12 hours after SCI in the mild (50 kdyn), moderate (70 kdyn) and severe (90 kdyn) SCI groups, as determined by histological quantification. *P < 0.05, ANOVA with the Tukey-Kramer post-hoc test (n = 7 mice per group). Scale bars (**A**): 500 μm; insets: 100 μm.

According to the histological findings of neutrophil infiltration in the injured spinal cord, we hypothesized that the number of infiltrating neutrophils may indicate the functional prognosis after SCI. We examined the number of infiltrating neutrophils in the injured spinal cord, and compared the number of neutrophils in the mild, moderate and severe SCI groups. Using flow cytometry, we counted the number of cells in the CD11b^high^/Gr-1^high^ cell subset, a commonly used population to detect neutrophils^17^,^18^, both in the spinal cord and the peripheral blood at 12 hours after SCI (Fig. 4A,B). The quantitative analysis revealed that the severity of SCI correlated with the number of infiltrating neutrophils in the injured spinal cord, whereas the number of infiltrating neutrophils in the peripheral blood was comparable among the three SCI groups (Fig. 4C,D). We then confirmed the infiltration of PMN-positive neutrophils in the injured spinal cord in the three groups by histological analysis and counted the number of infiltrating neutrophils. Consistent with the results of flow cytometry, the number of PMN-positive neutrophils in the injured spinal cord correlated with the severity of SCI (Fig. 5A,B). Because infiltrating peripheral blood cells include several cell types other than neutrophils^15^, we further examined whether other blood-derived cells were found in the injured spinal cord at 12 hours after SCI. Immunofluorescence staining with antibodies against PMN and CD45 (leukocyte common antigen) showed that infiltrating blood cells in the epicenter of the lesion were primarily composed of PMN-positive/CD45-positive neutrophils. We also found a few other cell types (PMN-negative/CD45-positive) in the injured spinal cord (Fig. S2). These findings indicate that the different numbers of infiltrating neutrophils with differently given injury severity could be a useful marker for predicting the functional prognosis after SCI.

### *In vivo* bioluminescence imaging predicts the functional prognosis after SCI

**Figure 6:**
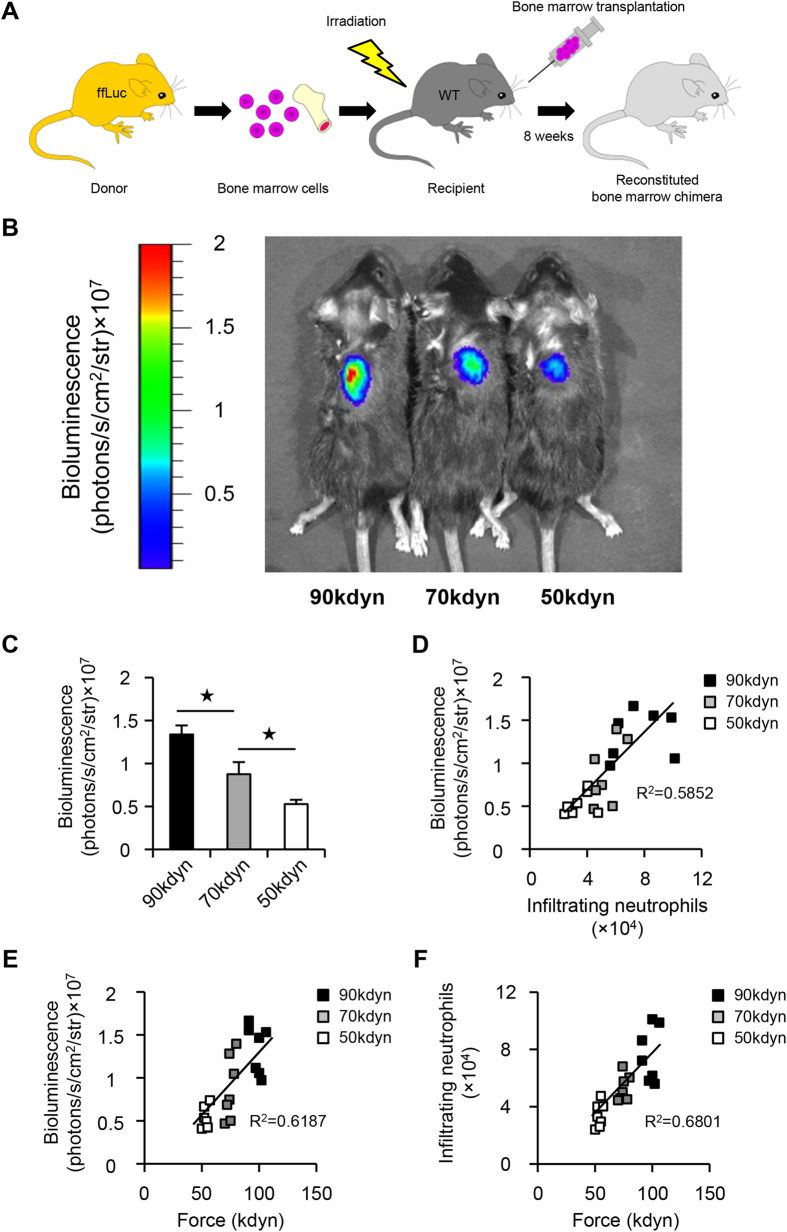
*In vivo* bioluminescence imaging predicts the functional prognosis after SCI. (**A**) A schematic illustration of the generation of bone marrow chimeric mice. Whole bone marrow was harvested from a firefly luciferase (ffLuc) transgenic mouse donor. Bone marrow cell transplantation was performed, and the reconstituted bone marrow chimeric mice were analyzed at eight weeks after transplantation. (**B**) Representative pictures of bioluminescence imaging of the injured spinal cord at 12 hours after SCI in the mild (50 kdyn), moderate (70 kdyn) and severe (90 kdyn) SCI groups. (**C**) The bioluminescence signal intensity in the injured spinal cord correlated with the severity of SCI. *P < 0.05, ANOVA with the Tukey-Kramer post-hoc test (n = 7 mice per group). (**D**) The correlation between the number of infiltrating neutrophils as measured by flow cytometry and the bioluminescence signal intensity as measured by *in vivo* imaging (n = 7 mice per group, P < 0.0001, Spearman’s rank correlation coefficient). (**E**) The correlation between the severity of SCI and the bioluminescence signal intensity (n = 7 mice per group, P < 0.0001, Spearman’s rank correlation coefficient). (**F**) The correlation between the severity of SCI and the number of infiltrating neutrophils (n = 7 mice per group, P < 0.0001, Spearman’s rank correlation coefficient).

Next, to detect the bioluminescence signals from infiltrating blood cells in the injured spinal cord by *in vivo* bioluminescence imaging, we developed a bone marrow chimeric mouse model in which peripheral blood cells selectively express luciferase (Fig. 6A). In this model, we successfully detected the intensity of bioluminescence signals from the injured spinal cord at 12 hours after SCI (Fig. 6B). In addition, to determine whether the *in vivo* bioluminescence imaging of infiltrating blood cells could predict the functional prognosis after SCI, we compared the bioluminescence signal intensity on *in vivo* imaging in the mild, moderate and severe SCI groups. We found that the bioluminescence signal intensity in the injured spinal cord correlated with the severity of SCI (Fig. 6C). We further compared the bioluminescence signal intensity with the number of infiltrating neutrophils by flow cytometry and revealed that there was a strong linear relationship between these two factors (Spearman’s rank correlation coefficient: bioluminescence vs. infiltrating neutrophils, R^2^ = 0.5852, P < 0.0001) (Fig. 6D). Furthermore, there was also a strong linear relationship between the severity of SCI and the bioluminescence signal intensity, as well as between the severity of SCI and the number of infiltrating neutrophils (Spearman’s rank correlation coefficient: severity vs. bioluminescence, R^2^ = 0.6187, P < 0.0001; severity vs. infiltrating neutrophils, R^2^ = 0.6801, P < 0.0001) (Fig. 6E,F). Taken together, these results demonstrate that *in vivo* imaging of the injured spinal cord is suitable for predicting the functional prognosis at 12 hours after SCI.

## Discussion

In this study, we reported three significant findings. First, *in vivo* bioluminescence imaging of the injured spinal cord may potentially predict the functional prognosis at 12 hours after SCI. Second, neutrophils were the predominant cell type to infiltrate the injured spinal cord at 12 hours after SCI. Third, the number of infiltrating neutrophils, as well as the bioluminescence signal intensity in the injured spinal cord, correlated with the severity of SCI. We herein propose a novel approach for the classification of SCI patients according to the severity of SCI as assessed by *in vivo* bioluminescence imaging. The analysis of this imaging method could also provide a basis for the development of therapeutic protocols that are tailored according to the severity of SCI.

Although many studies have shown the availability of serum biochemical markers to predict the functional prognosis after SCI, there is a practical limitation in applying these markers to SCI patients. The subjects in previous SCI clinical trials potentially included patients with complications, which could distort the biochemical data. Indeed, Pelinka *et al*. reported that the serum S100β and GFAP levels, which had been previously established as useful biochemical markers after central nervous system (CNS) injury, were unreliable for predicting the functional prognosis in patients with concomitant multiple trauma or hemorrhagic shock after CNS injury^19^. Thus, in order to predict the functional prognosis using such biochemical data, the SCI patients with severe complications should be excluded from clinical trials. These findings also highlight the importance of the *in vivo* non-invasive imaging method, which enabled us to predict the functional prognosis after SCI, irrespective of complications as this imaging instrument focuses only on the signals from the site of the injury.

Other imaging modalities, such as MRI and DTI, are useful for non-invasively predicting the functional prognosis after SCI; however, the *in vivo* bioluminescence imaging method has several advantages over these modalities. Bioluminescence imaging simplifies the process of capturing images and involves an extremely short acquisition time (within 60 seconds per image)^11^,^20^. The long acquisition time of MRI means that in most SCI patients it can only be applied once in the acute phase of SCI. More importantly, the MRI findings in the acute phase of SCI display a high degree of variability according to the timing of imaging. Notably, within 24 hours after SCI, the oxidation status of erythrocytes, which has the greatest influence on the T2 signal intensity in MRI images, changes drastically every few hours^21^. Thus, the prolonged acquisition time in MRI may make it unsuitable for predicting the functional prognosis after SCI^22^. Bioluminescence imaging allows for multiple images to be captured in a short period of time and enables the observation of time-dependent pathological changes in SCI, especially in the acute phase.

The first hematogenous inflammatory cell to arrive at the site of injury is the neutrophil^16^. In this study, we conceived the idea of applying the bioluminescence imaging method for predicting the functional prognosis after SCI because the number of infiltrating neutrophils is dependent on the severity of SCI. The direct link between infiltrating neutrophils in the early phase of SCI and the locomotor function in the chronic phase of SCI remains controversial^23^. Neutrophil infiltration participates in the release of protective cytokines that promote axonal sparing and tissue repair^24^,^25^. Conversely, neutrophil infiltration is also related to inflammation, neural cells death, secondary damage, and scar formation^26^,^27^. These mechanisms are associated with the spinal physiology and, as a result, locomotor recovery. According to the proportional relationship between the number of infiltrating neutrophils and the severity of the injury, infiltrating neutrophils might have a detrimental effect on the functional prognosis after SCI.

Previous reports showed that resident macrophages, peripheral macrophages, and astrocytes proliferate at 3 days after SCI, and their numbers significantly increased following SCI^28^,^29^. These findings could be valuable for the development of a bioluminescence application for the prediction of the functional prognosis after SCI. However, at 3 days after SCI, only the functional evaluation sufficiently enabled us to predict the function prognosis after SCI^2^,^30^,^31^, indicating that bioluminescence imaging should be applied before 3 days of SCI to predict the functional prognosis after SCI. As we described in this study, bioluminescence imaging of blood-derived cells enabled us to predict the functional prognosis at 12 hours after SCI. The infiltrating blood-derived cells in the lesion area were primarily composed of neutrophils; therefore, bioluminescence imaging of neutrophils could be more useful than that of resident macrophages, peripheral macrophages, or astrocytes in order to establish a method to predict the functional prognosis after SCI.

Although we observed that the number of infiltrating neutrophils at 12 hours after SCI correlated with the severity of the injury, we observed a comparable number of neutrophils in the peripheral blood among the three groups of SCI (Fig. 4). Previous studies showed that neutrophilic mobilization from bone marrow into the peripheral blood occurs a few hours after the transient change of neutrophilic distribution^32^,^33^,^34^. In this study, we did not compare the number of neutrophils in the peripheral blood between the mice with SCI and those without SCI. Even though differently given SCI severity may have influenced the number of neutrophils in the peripheral blood in the hyperacute phase of SCI, neutrophils in the peripheral blood could be rapidly rearranged in a regulated manner. Indeed, in SCI patients, the number of circulating peripheral neutrophils was comparable between the SCI group and the uninjured group, whereas the number of infiltrating neutrophils increased in the injured spinal cord^16^,^35^. These previous findings in combination with our results suggest that the number of neutrophils in the injured spinal cord is more reliable than those in the peripheral blood for predicting the functional prognosis after SCI.

In our previous report, we showed that the most influential factor on the functional prognosis is the severity of paralysis at admission in SCI patients^3^, indicating that the functional prognosis is dependent on the severity of the initial injury in humans, as well as animals. Although the severity of paralysis influences the functional prognosis, most SCI patients undergo spinal shock in the hyperacute phase of SCI (within 24 hours after SCI), irrespective of the severity of the injury^2^; therefore, predicting the functional prognosis during the hyperacute phase of SCI is extremely difficult. We thus focused on the number of neutrophils in the hyperacute phase of SCI, because the neutrophil is the first hematogenous inflammatory cell to arrive at the site of injury^16^, and we examined whether the severity of SCI is dependent on the number of infiltrating neutrophils in mice. In SCI patients, neutrophils were reported to infiltrate the injured spinal cord in the hyperacute phase of SCI^16^, which was consistent with our results in mice. These findings support the notion that bioluminescence imaging of neutrophils could be useful for SCI patients. Because there would be great differences between individual patients regarding inflammatory tolerance, neuronal protection, and regeneration ability, our single study in mice could not sufficiently provide assurance that this imaging method is applicable for predicting the functional prognosis in SCI patients. Therefore, future studies investigating the link between the severity of SCI injury and the pathophysiology in SCI patients could provide additional information for establishing a bioimaging method to predict the functional prognosis after SCI in humans.

We successfully detected bioluminescence signals from neutrophils in the injured spinal cord of mice by transplanting bone marrow cells expressing luciferase into wild-type mice. However, this methodology is obviously not applicable to human patients with SCI; one major obstacle to applying this modality is that human neutrophils do not endogenously express luciferase. Several reports have demonstrated that endogenous bioluminescence signals from neutrophils could be detected using the luminol reaction: hypochlorous acid (HOCl), which is produced by myeloperoxidase (MPO) in neutrophils, oxidizes luminol and produces light^20^,^36^. In parallel with the present study, we attempted to detect endogenous bioluminescence signals derived from neutrophils in the injured spinal cord through the luminol reaction; however, we failed to identify any signals from the injured spinal cord of wild-type mice without the transplantation of luciferase-positive bone marrow cells. This failure is presumably due to the insufficiency of the endogenous signal intensity from neutrophils. In contrast to our findings, Zhang *et al*. recently succeeded in detecting endogenous signals from the neutrophils in deep tissues with the use of chemiluminescence resonance energy transfer (CRET)^37^. In addition, Shuhendler *et al*. presented a nanosensor to evaluate MPO activity through the combination of *in vivo* bioluminescence imaging and a newly synthesized semiconducting polymer nanoparticle (SPN)^38^. On the basis of these studies, an analytical method for detecting endogenous bioluminescence from the injured spinal cord should be developed in order to utilize *in vivo* bioluminescence imaging in SCI patients.

The intensities of the bioluminescence signal are affected by many factors, such as the level of luciferase expression, the concentration of luciferin injected, and the metabolic status of the cells, which may confound the quantitative results acquired from consecutive imaging series or different tissue types^39^. In addition, the efficiency of light transmission is limited by light scattering inside the tissue^40^. Thus, the detection sensitivity is a potential limitation of bioluminescence imaging for the prediction of the functional prognosis after SCI. In the present study, we examined only three defined SCI severities and did not demonstrate the sensitivity of this system. A careful design of the imaging method is necessary to minimize the effect of these confounding factors. More precise detection with bioluminescence imaging should be developed for enhancing the predictive power to predict the functional prognosis after SCI.

In the present study, we proposed a wide variety of applications of bioluminescence imaging to investigate not only neurodegenerative diseases, but also traumatic CNS injury. Bioluminescence imaging has previously been used to analyze the pathogenesis of neurodegenerative diseases, such as multiple sclerosis, Parkinson’s disease and Alzheimer’s disease^41^,^42^. The combination of bioluminescence imaging and transgenic mice expressing luciferase under the doublecortin (DCX) promoter, which is a specific marker of neuronal progenitor cells, allowed for monitoring of neurogenesis in living mice with experimental multiple sclerosis^43^. Furthermore, bioluminescence imaging has been shown to be capable of predicting the onset of Alzheimer’s disease in mice^44^. In these studies, bioluminescence signals were detected in resident neural cells. Moreover, we succeeded in detecting bioluminescence signals from non-resident infiltrating neutrophils, which were triggered by traumatic injury. Our findings represent a significant step in the elucidation of the pathophysiology of SCI.

In conclusion, we successfully established an accurate method for predicting the functional prognosis at 12 hours after SCI using *in vivo* bioluminescence imaging. We believe that this method could therefore contribute to the development of tailored therapeutic approaches that can be applied to SCI patients. Further investigation to detect endogenous bioluminescence signals in the injured spinal cord will be necessary for the future application of this modality in SCI patients.

## Materials and Methods

### Animals

Adult 8- to 12-week-old female C57BL/6 wild-type mice, CAG-EGFP transgenic mice and ffLuc-cp156 transgenic mice were used in this study. The background strain of all the mice used in this study was C57BL/6. The ffLuc-cp156 protein consists of a fusion protein of firefly luciferase and circularly permuted Venus protein^45^. The ffLuc-cp156 mice were generated using a construct composed of a CAG-promoter driven combination cassette bearing a neomycin-coding sequence sandwiched by two loxP sequences and the ffLuc-cp156 gene. To yield a mouse line that ubiquitously produced ffLuc-cp156, a C57BL/6 line carrying the reporter gene (CAG-neo^loxp/loxp^-ffLuc) was crossed with a C57BL/6 line which ubiquitously expressed Cre recombinase (CAG-Cre).

### Surgical procedures

Mice were anesthetized via an intraperitoneal injection of pentobarbital (75 mg/kg). After laminectomy at the 9^th^ thoracic level, the dorsal surface of the dura matter was exposed and SCI was induced using a commercially available SCI device (Infinite Horizons Impactor, Precision Systems Instrumentation, Lexington, KY). This device creates a reliable contusion injury by rapidly applying a force-defined impact with a stainless steel-tipped impounder^46^. Injury with the impactor was performed at force settings of 50 kdyn, 70 kdyn, or 90 kdyn (n = 84 mice). After injury, the overlying muscles were sutured, and the skin was closed with nylon sutures. During recovery from anesthesia, the animals were placed in a temperature-controlled chamber until thermoregulation was re-established. All surgical procedures and experimental manipulations were approved by the Committee of Ethics on Animal Experiment in Faculty of Medicine, Kyushu University. Experiments were conducted in accordance with the institutional guidelines and regulations for animal experiments.

### Behavioral analysis

The motor functions were evaluated using the locomotor open-field rating scale of the Basso Mouse Scale (BMS) locomotor rating scales^47^. A team of two experienced examiners evaluated each animal for four minutes and assigned an operationally-defined score for each hindlimb in an open field, which was a molded-plastic circular enclosure with a smooth, nonslip floor. The assessment of functional recovery began at postoperative 12 hours. Each mouse was assessed at 12 hours, 1day, 4 days and 7 days postoperatively and weekly thereafter until 6 weeks. In order to characterize the functional change in the BMS score in the acute phase of SCI, time-dependent recovery as assessed by the BMS score in mice in the 50 kdyn, 70 kdyn, and 90 kdyn SCI groups was calculated between 0–12 hours, 12–24 hours, and 1–4 days postoperatively.

### Bioluminescence imaging

Bioluminescence imaging was performed using the Xenogen-100 system following a previously published protocol^48^. For *in vivo* imaging, the mice were given a subcutaneous injection of D-luciferin (2 mg/kg body weight) at the site of injury, and serial images were acquired from 15 minutes after the administration until the maximum intensity was obtained. Digital images were recorded and analyzed using the Living Image Software program (Caliper Life Sciences). We analyzed images with a consistent region-of-interest (ROI), which was placed over the injured area (2 mm × 2 mm) to calculate the bioluminescent signal.

### Bone marrow chimeras

Bone marrow chimeras were prepared as previously described^49^. In brief, chimeras were prepared by subjecting gender-matched recipient wild-type mice to lethal whole-body irradiation (10 Gy). The recipient mice were then reconstituted with 1 × 10^7^ bone marrow cells derived from CAG-EGFP mice or ffLuc-cp156 mice. Chimeric mice were used eight weeks after bone marrow transplantation.

### Flow cytometry

Blood samples and spinal cord samples (6.0 mm in length, centered on the lesion) were prepared for flow cytometry as previously described^14^. For the blood samples, red blood cells were removed with a hypotonic lysis buffer (17 mmol/L Tris-HCl, pH 7.2; 100 mmol/L NH4Cl), and the resulting suspension was pelleted by centrifugation and washed twice in PBS. Spinal cord samples were dissected and mechanically dissociated with collagenase (175 U/mL, Invitrogen) for 30 minutes at 37 °C. Cells were washed in Dulbecco’s modified Eagle’s Medium (DMEM) containing 10% fetal bovine serum, and passed through a 40 μm nylon cell strainer (BD Biosciences) to isolate tissue debris from the cell suspension. The resulting suspension from the spinal cord and blood samples was centrifuged and the pellet was re-suspended and incubated for 30 minutes on ice with the fluorescent antibodies. Samples were stained with anti-CD45, anti-CD11b and anti-Gr-1 purchased from BioLegend. Prior to the analysis, propidium iodide was added to determine cell viability. The samples were analyzed on a FACSAria II flow cytometer (BD Biosciences), while the data were analyzed using the FACSDiva software program (BD Biosciences).

### Histopathological examination

After the mice were transcardially fixed with 4% paraformaldehyde, the spinal cord was removed, dehydrated and embedded in OCT compound. The frozen tissues were cut in the sagittal plane into 16 μm sections. For immunostaining, the sections were stained with primary antibodies against PMN (neutrophil marker, 1:200, rat, Serotec), Iba1 (monocyte/macrophage marker, 1:200, rabbit, Wako), or laminin (basement membrane marker, 1:200, rabbit, Sigma-Aldrich). The sections were then incubated with Alexa Fluor-conjugated secondary antibodies (1:200, Invitrogen). Nuclear counterstaining was performed using Hoechst 33342 (Molecular Probes).

### Image acquisition and quantitative analysis

All images were obtained using a BZ-9000 digital microscope system (Keyence). For the quantification of infiltrating neutrophils, we performed immunostaining with anti-neutrophil antibody in each sagittal section at 150 μm intervals from side to side and captured 120 regions with 100x magnification in each section to reconstruct a 2 mm section, which occupied a region of 1000 × 900 × 2000 μm in the dorsoventral, mediolateral, and rostrocaudal dimensions, respectively, in each mouse (n = 7 in each group). The algorithms for counting the number of infiltrating neutrophils were provided by the Dynamic Cell Count BZ-H1C software program (Keyence), which selectively counted immunopositive particles in sizes ranging from 5 μm to 15 μm in both the X- and Y- dimensions and automatically eliminated spurious particles.

### Statistical analysis

For multiple comparisons of the differences in the number of neutrophils or in the bioluminescence signals among the SCI groups, one-way factorial analysis of variance (ANOVA) with a post-hoc Tukey-Kramer test was performed. For multiple comparisons of the differences in the BMS scores among the three SCI groups as well as in the increment in the BMS score among the three SCI groups over time, two-way repeated-measures ANOVA with a post-hoc Tukey-Kramer test was performed. Correlational analyses were performed using Spearman’s rank correlation coefficient. In all statistical analyses, significance was defined as P < 0.05. The values for groups are presented as the average ± SEM. All statistical analyses were carried out using the JMP software program (version 9; SAS Institute).

## Additional Information

**How to cite this article**: Yokota, K. *et al*. The feasibility of *in vivo* imaging of infiltrating blood cells for predicting the functional prognosis after spinal cord injury. *Sci. Rep.* **6**, 25673; doi: 10.1038/srep25673 (2016).

## Supplementary Material

Supplementary Information
