# Supplementary material for: The feasibility of in vivo imaging of infiltrating blood cells for predicting the functional prognosis after spinal cord injury
Source: Sci Rep. 2016 May 9;6:25673. doi: 10.1038/srep25673 (PMC4860707; doi:10.1038/srep25673)
Supplement: Supplementary Information [file srep25673-s1.pdf]

Supplementary Information

The feasibility of in vivo imaging of infiltrating blood cells for predicting the functional prognosis after spinal cord injury

Kazuya Yokota, Takeyuki Saito, Kazu Kobayakawa, Kensuke Kubota, Masamitsu Hara, Masaharu Murata, Yasuyuki Ohkawa, Yukihide Iwamoto and Seiji Okada

The PDF file includes

Figure S1. Newly formed blood vessels are rarely observed within 12 hours after SCI.

Figure S2. Infiltrating blood cells in the epicenter of the lesion are primarily composed of neutrophils at 12 hours after SCI.

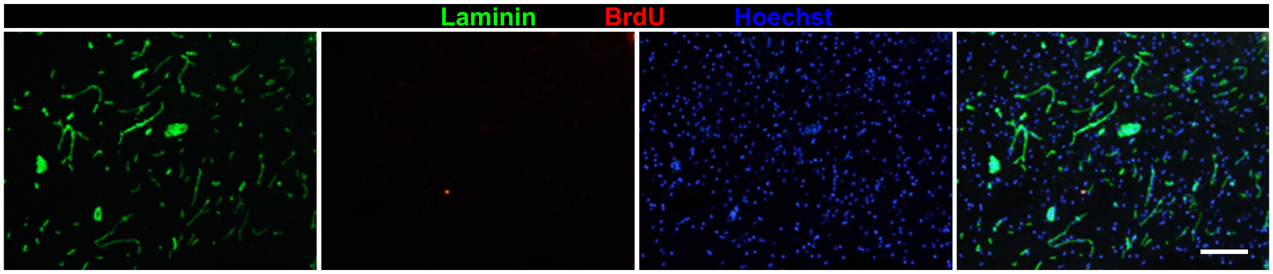

Figure S1. Newly formed blood vessels are rarely observed within 12 hours after SCI

An immunohistochemical analysis of the injured spinal cord at 12 hours after SCI with ;aminin (green), BrdU (red), and Hoechst (blue) staining.

Scale bar: 100 µm.

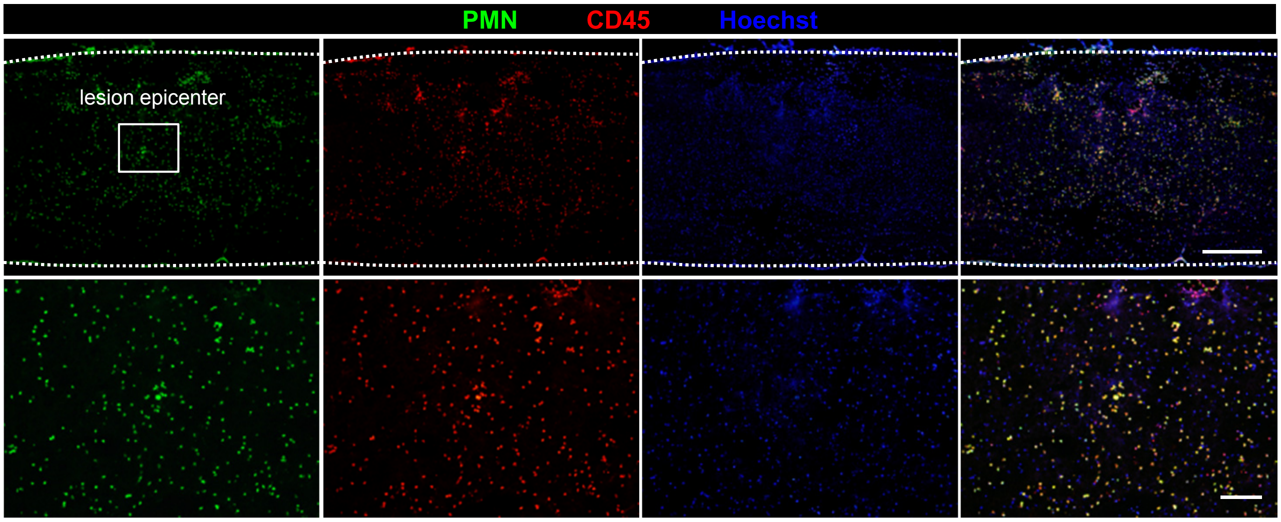

Figure S2. Infiltrating blood cells in the epicenter of the lesion are primarily composed of neutrophils at 12 hours after SCI.

An immunohistochemical analysis of the injured spinal cord at 12 hours after SCI with PMN (green), CD45 (red) and Hoechst (blue) staining.

Scale bars: 500 µm; inset: 100µm.
